# Saliva cortisol in relation to aircraft noise exposure: pooled-analysis results from seven European countries

**DOI:** 10.1186/s12940-019-0540-0

**Published:** 2019-11-27

**Authors:** Clémence Baudin, Marie Lefèvre, Jenny Selander, Wolfgang Babisch, Ennio Cadum, Marie-Christine Carlier, Patricia Champelovier, Konstantina Dimakopoulou, Danny Huithuijs, Jacques Lambert, Bernard Laumon, Göran Pershagen, Töres Theorell, Venetia Velonaki, Anna Hansell, Anne-Sophie Evrard

**Affiliations:** 1 Univ Lyon, Université Claude Bernard Lyon1, ifsttar, umrestte, umr t_9405, Cité des Mobilités, 25 avenue François Mitterrand, F-69675 Bron, France; 2 Now at: Technical Agency for Information on Hospital Care, Lyon, France; 3 Institute of Environmental Medicine, Karolinska Institute, Stockholm, Sweden; 4 Federal Environment Agency, Berlin, Germany; 5 Environmental Health Unit, Agency for Health Protection, Pavia, Italy; 6 Hospices Civils de Lyon GH Sud CBAPS Laboratoire de Biochimie, Pierre Bénite, France; 7 Currently retired, Bron, France; 8 IFSTTAR, Planning, Mobilities and Environment Department, Dynamics of Mobility Changes Team, Bron, France; 9 Department of Hygiene, Epidemiology and Medical Statistics Faculty of Medicine, National and Kapodistrian, University of Athens, Athens, Greece; 10 National Institute of Public Health and Environmental Protection, Bilthoven, the Netherlands; 11 IFSTTAR, Transport, Health and Safety Department, Bron, France; 12 Institute of Environmental Medicine, Karolinska Institute, Stockholm, Sweden; 13 Stress Research Institute, Faculty of Social Sciences, Stockholm University, Stockholm, Sweden; 14 Nurses School, National and Kapodistrian, University of Athens, Athens, Greece; 15 Centre for Environmental Health and Sustainability, University of Leicester, Leicester, UK

**Keywords:** Epidemiology, Aircraft noise exposure, Saliva cortisol

## Abstract

**Background:**

Many studies have demonstrated adverse effects of exposure to aircraft noise on health. Possible biological pathways for these effects include hormonal disturbances. Few studies deal with aircraft noise effects on saliva cortisol in adults, and results are inconsistent.

**Objective:**

We aimed to assess the effects of aircraft noise exposure on saliva cortisol levels and its variation in people living near airports.

**Methods:**

This study focused on the 1300 residents included in the HYENA and DEBATS cross-sectional studies, with complete information on cortisol sampling. All the participants followed a similar procedure aiming to collect both a morning and an evening saliva cortisol samples. Socioeconomic and lifestyle information were obtained during a face-to-face interview. Outdoor aircraft noise exposure was estimated for each participant’s home address. Associations between aircraft noise exposure and cortisol outcomes were investigated a priori for male and female separately, using linear regression models adjusted for relevant confounders. Different approaches were used to characterize cortisol levels, such as morning and evening cortisol concentrations and the absolute and relative variations between morning and evening levels.

**Results:**

Statistically significant increases of evening cortisol levels were shown in women with a 10-dB(A) increase in aircraft noise exposure in terms of LA_eq, 16h_ (exp(β) = 1.08; CI95% = 1.00–1.16), L_den_ (exp(β) = 1.09; CI95% = 1.01–1.18), L_night_ (exp(β) = 1.11; CI95% = 1.02–1.20). A statistically significant association was also found in women between a 10-dB(A) increase in terms of L_night_ and the absolute variation per hour (exp(β) = 0.90; CI95% = 0.80–1.00). Statistically significant decreases in relative variation per hour were also evidenced in women, with stronger effects with the L_night_ (exp(β) = 0.89; CI95% = 0.83–0.96) than with other noise indicators. The morning cortisol levels were unchanged whatever noise exposure indicator considered. There was no statistically significant association between aircraft noise exposure and cortisol outcomes in men.

**Conclusions:**

The results of the present study show statistically significant associations between aircraft noise exposure and evening cortisol levels and related flattening in the (absolute and relative) variations per hour in women. Further biological research is needed to deepen knowledge of the pathway between noise exposure and disturbed hormonal regulation, and specially the difference in effects between genders.

## Introduction

Aircraft noise exposure represents a major issue for public health policies. Impacts on human health are of growing concern, and many adverse effects have been evidenced [1]. Extensive information is available to quantify the burden of disease from aircraft noise exposure associated with annoyance, sleep disturbances, cardiovascular disease including hypertension [2–7], and altered cognitive performance among children [8, 9]. A proposed biological process is the release of stress hormones with noise exposure, leading to disruption of hormonal rhythms by activating the Sympathetic-Adrenal-Medullary (SAM) axis and the Hypothalamic-Pituitary-Adrenal (HPA) axis [10, 11]. Cortisol can be viewed as a stress indicator, and is easy to measure non-invasively [12]. Its concentration may be a clinical indicator of disturbed HPA axis activity, and therefore can be used to assess chronic stress effects due to noise exposure [13]. Specific roles of cortisol include regulation of blood glucose levels, lipolysis, immune suppression, and regulation of blood pressure [14]. The cortisol secretion follows a circadian rhythm in the absence of stimuli: levels decline slowly throughout the day, from a peak in the early morning (20–30 min after awakening) to a nadir in the evening [15]. Cortisol can easily be measured in saliva and this reliably reflects the serum-free cortisol concentration [16].

The majority of the studies have focused on average levels of cortisol at specific times of the day [17, 18]. However, when subjects are chronically exposed to high levels of energy mobilization after exposure to a stressor, the regulation of the cortisol response could be disturbed. This may be in the form of inability to lower cortisol during the calmer part of the day with no decrease in cortisol when the subject is expected to fall asleep. After long intensive periods without possibility to recuperate, the subject may develop a low flat curve, which is associated with inability to mobilize cortisol when it is needed [19]. Several studies showed an association between long-term stress exposure and a flattening of the diurnal cortisol rhythm across the day [20–22]. The variability of cortisol during the day could therefore be used as an indicator of a disturbed HPA axis regulation.

Many studies have been carried out on the relationship between noise exposure and cortisol levels, but conclusions are still unclear [12]. Biological responses may differ depending on the source (occupation, road traffic, rail or air) and characteristics of the study population (gender, age) [23]. Studies on aircraft noise exposure and cortisol levels have mainly focused on children’s populations. The main studies to date on adults were a subset of HYENA (HYpertension and Exposure to Noise near Airports) [24] and DEBATS (Discussion on the health effects of aircraft noise) [5] projects. The HYENA study analysis found increases in morning saliva concentrations with aircraft noise exposure in women only [17], while DEBATS found higher evening but not morning cortisol in both men and women [25].

The present HYDE (**HY**ENA + **DE**BATS) project aims to combine both HYENA and DEBATS datasets in order to elucidate the effects of aircraft noise exposure on saliva cortisol levels. The HYENA study included persons living near one of seven major European airports [London Heathrow (United Kingdom), Berlin Tegel (Germany), Amsterdam Schiphol (the Netherlands), Stockholm Arlanda and Bromma (Sweden), Milan Malpensa (Italy), and Athens International Airport Eleftherios Venizelos (Greece) Airports]. Specifically, the HYDE present project added to the HYENA study the three French airports included in the DEBATS study: Lyon Saint Exupéry, Toulouse-Blagnac, and Paris-Charles de Gaulle – the latter being a major European airports in term of passenger numbers [26]. Combination of HYENA and DEBATS enabled a higher number of participants to be included in the analyses, resulting in an increase in statistical power, and extending the scope of the results. As gender differences in production of corticosteroid-binding globulin have been shown [27], the higher number of participants in the HYDE study allowed us to investigate the association between aircraft noise exposure and cortisol secretion for male and female separately. Moreover, the HYDE study also provided an opportunity to consider a new approach based on the relative variation in cortisol levels per hour taking into account time between measurements as well as morning and evening levels.

## Methods

### Study population

The main HYENA study included participants aged 45–70 at the time of the interview, living near one of seven major European airports in six countries. Participants were selected at random from available registers (e.g. registration office, electoral roll, health service). Data were collected on 4861 participants between 2004 and 2006 [24]. The sub sample for saliva sampling was randomly selected from the participants in the main study. Priority was given to participants with the highest and lowest levels of exposure to aircraft noise in each country. The lowest level of exposure corresponds to < 50 dB(A) in terms of LAeq,24 h in all countries. The highest level of exposure was not the same in all countries: it varies from > 60 dB(A) in Italy, Greece, the Netherlands and Sweden to > 65 dB(A) in Germany and > 69 dB(A) in the UK. With the purpose to recruit 84 subjects from each of the six participating countries, complete information for cortisol samples were finally provided for a total number of 473 participants.

The main DEBATS study included people over 18 years of age at the time of the interview, living in the study area around three French international airports [6]. Participants were selected at random from a phone directory, based on their address in the study area and contacted by phone. Cortisol sampling were collected on all 1244 participants (549 men and 695 women) in 2013 [5]. Complete information for cortisol samples were provided for 1199 of them.

In both studies, all the participants responded to a questionnaire during a face-to-face interview at their place of residence. This questionnaire collected information on demographic and socioeconomic characteristics, lifestyle factors including smoking habits, alcohol consumption, and physical activity, personal medical history in terms of sleep disturbance, cardiovascular diseases, anxiety, depressive disorders, medication use, and annoyance due to noise exposure. Blood pressure, anthropometric measurements (weight and height), and saliva samples (cortisol concentrations) were recorded.

As cortisol levels are related to the circadian rhythm, which is sensitive to individual schedule, participants with a typical working (shift workers, working at night) and/or typical sleeping patterns were excluded from the pooled analyses (*N* = 341). Other exclusions were made for participants with missing information on cortisol samples (concentrations, dates, and times) and those with 24 h or more between the both saliva samplings.

**Fig. 1:**
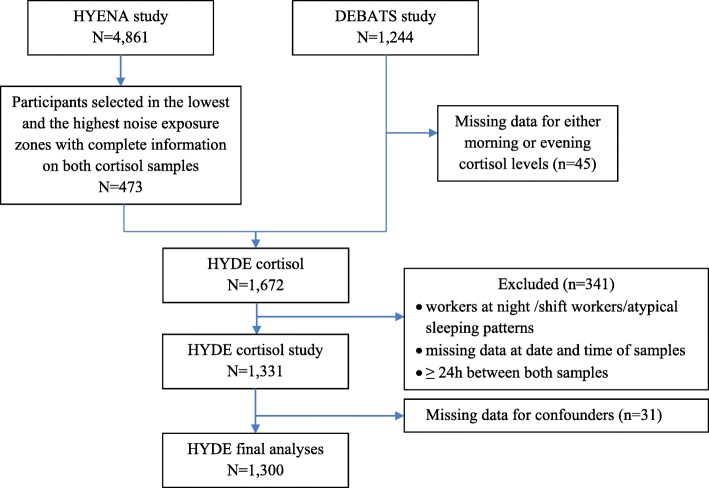
Flow chart of HYDE cortisol participants

The final pooled analyses were carried out on N_HYDE_ = 1300 participants (359 from HYENA and 941 from DEBATS, including 555 men and 745 women) who had completed information for all the covariates included in the model (Fig. 1).

Each centre’s ethical committee gave study approval and each participant provided written informed consent.

### Cortisol measurements

In HYENA, participants received a kit with test tubes (Sarstedt, Nümbrecht, Germany) and instructions the week before the interview. Samples had to be collected the day before the interview and given to the staff during the interview. Tubes were sent to, centrifuged and frozen in laboratory in each of the participating countries. When all samples had been received in each country, saliva tubes were sent to Karolinska Institutet laboratory (Stockholm, Sweden) for analysis. Cortisol levels in saliva were determined by the Spectria cortisol coated tube radioimmunoassay kit (Orion Diagnostica, Espoo, Finland). In DEBATS, participants received a kit with test tubes (Sarstedt, Nümbrecht, Germany) and instructions during the interview. Samples had to be collected and given to the staff in the days following the interview. Tubes were sent to, and frozen in a laboratory in Lyon (France). Cortisol levels in saliva were determined by the cortisol saliva ELISA kit (IBL international, Hamburg, Germany).

Instructions for the procedure in measurements were similar in both studies. Participants were requested to collect a sample 30 min (in HYENA) (corresponding usually to the peak in cortisol concentration) or immediately (in DEBATS) after awakening, and another one just before going to bed in the evening (which usually coincides with the nadir in cortisol concentration). Tooth brushing, smoking, and food and drink intake were to be avoided 30 min before each sampling. Each of the test tubes included a small cotton swab that participants were instructed to put in their mouth and to chew until it was completely soaked in saliva. Then, the swab had to be placed in the test tube and stored in a fridge, after writing date and time on the label of the tube. All samples were analysed simultaneously in duplicate.

### Aircraft noise exposure assessment

Aircraft noise exposure was estimated at the place of residence of the participants, in front of the buildings. For all countries except the UK, aircraft noise levels were provided from the “Integrated Noise Model” (INM) [28]. The INM is an internationally well-established computer model that evaluates aircraft noise impacts in the vicinity of airports and outputs noise contours for an area. The UK used the national Aircraft Noise Contour Model (ANCON v 2) [29], similar to the INM model. The INM and ANCON models require the following input data: actual measurements, the approach and departure routes or flight tracks, the traffic upon them in terms of the numbers of different aircraft types, the dispersion of individual flight tracks, the average flight profiles (of height, noise emission and speed) of the different aircraft types [30, 31].

Outdoor aircraft noise exposure was assessed in 1-dBA intervals for each participant by linking his/her home address to the noise contours using geographical information systems (GIS) methods. Four noise indicators referring to three different periods of the day were derived and used for the statistical analyses: L_den_, L_Aeq,24h_, L_Aeq_,_6h–22h_, and L_night_. The L_den_ is an indicator for the 24 h period among others defined in the EU directive 2002/49 [32] relating to the assessment and management of environmental noise. It is defined as the weighted average of sound levels during daytime (06:00 to 18:00 or 7:00 to 19:00, depending on the country), evening (18:00 to 22:00 or 19:00 to 23:00), and night-time (22:00 to 6:00 or 23:00 to 7:00), where evening and night sound pressure levels received a 5 dB(A) and a 10 dB(A) penalty respectively to reflect the extra sensitivity to noise during the evening and the night. The L_Aeq,24h,_ L_Aeq_,_6h–22h_ and L_night_ correspond to average sound levels during the corresponding period of time.

### Annoyance due to aircraft noise

Aircraft noise annoyance was assessed using the ISO/Icben (International Commission on the Biological Effects of Noise) recommended question [33], both in HYENA and in DEBATS: “Thinking about the last 12 months when you are here at home, how much does aircraft noise bother, disturb or annoy you?”

Then, in HYENA, the standard numeric scale was used for night-time and daytime annoyance separately (range 0–10). In the present HYDE study, an average score between night-time and daytime score was calculated, and participants with an average score ≥ 8 were considered as being highly annoyed.

In DEBATS, the standard verbal scale was used with five possible answers: extremely, very, moderately, slightly or not at all. Extremely or very annoyed participants were considered as being highly annoyed.

### Noise sensitivity

In HYENA, noise sensitivity was assessed with the short-form of the Weinstein scale [34] including 10 items where people were asked to evaluate how much (from 1 to 6) they agreed with different statements about noise. One question concerned sensitivity to noise.

In DEBATS, the following 5-point question was used to assess noise sensitivity: “Regarding noise in general, compared to people around you, do you think that you are: much less sensitive than, or less sensitive than, or as sensitive as, or more sensitive, or much more sensitive than people around you?”

The sensitivity question in HYENA was assimilated to the one in DEBATS as follows: 1 corresponds to “much less sensitive”, 2 to “less sensitive”, 3 and 4 to “as sensitive”, 5 to “a little more sensitive” and 6 to “ much more sensitive”.

### Confounders

Information about major potential confounders were obtained from the face-to-face interview and were a priori included in the models: country, gender (dichotomous), age (continuous), BMI (continuous), smoking habits (five categories: non-smoker; ex-smoker; 1–10 units/day; 11–20 units/day; > 20 units/day), alcohol consumption (4 categories: teetotaller; 1–7 units a week; 8–14 units/week; > 14 units/week), physical activity (2 categories: no or a little; regular), and education level (coded as quartiles of number of years in education previously standardized by country means). Education level was included in the models as a possible confounder, as a proxy for income. Indeed, this variable was available in all countries, unlike the income that is forbidden to be collected in the UK.

### Statistical analysis

The HYENA and the DEBATS datasets were first pooled and harmonized according to common variables. Then, different outcomes were tested, as cortisol is a biological measure following a circadian rhythm. 1) We analysed the morning (C_1_) and the evening levels (C_2_) in cortisol separately (nmol. L^− 1^). 2) We investigated the average variation in cortisol per hour (nmol. L^− 1^.H^− 1^) between both samplings (calculated as (C_1_-C_2_) / (|T_1_-T_2_|) where T_1_ corresponds to sampling time for C_1_ and T_2_ corresponds to sampling time for C_2_). The variation in cortisol was firstly defined in the absolute difference between evening and morning saliva sample concentrations because cortisol levels are expected to decrease over the day. As time between both samplings varied between participants, the variation in cortisol was divided by the time in hours between the two samplings to enable comparisons. 3) We tested the average relative-variation in cortisol per hour between both samplings [(C_1_-C_2_)/C_1_] / (|T_1_-T_2_|). The variation in cortisol per hour was divided by the morning level as reference level, thus allowing for individual differences in cortisol levels and also for potential measurement differences between HYENA and DEBATS related to sampling equipment and laboratory analysis.

Each of these outcomes were firstly log-transformed to compensate for a non-normal distribution. Then, we analysed each outcome in relation to aircraft noise exposure using linear regression, adjusted for the confounders. As a literal interpretation for log-linear results, considering Y as a cortisol outcome, the expected value of Y is multiplied by exp.(β) for each 1-unit increase in X. Thus, to interpret results in our study, the given value exp.(β) is the multiplier to be applied to the considered cortisol outcome in order to get its expected value with a 10-dB(A) increase in noise level.

Studies reported consistently higher cortisol values with ELISA method (applied in the DEBATS study) than with RIA method (applied in the HYENA), for same samples. Compared to controls, RIA gave results much closer to the expected value than ELISA did [33]. Although measurements concerned only 10 samples, Baecher et al. (2013) published Passing and Bablock regression of salivary cortisol results reported by immunoassay systems related to a reference (35). Regression equations showed a strong linear relationship between ELISA and RIA methods: RIA = 0.92 (95% CI: 0.87–1.03) × ELISA-0.19 (95% CI: − 0.35 to − 0.04); r = 0.993. This last equation was used in sensitivity analyses to make the levels of cortisol concentrations between the two studies comparable.

As gender differences in production of corticosteroid-binding globulin have been shown [27], analyses were carried out for male and female separately. Moreover, in previous analyses carried out on HYENA participants, no substantial differences in effect of noise have been shown between countries [36, 37]. Therefore, the interaction term between country and noise exposure was tested but as it was not statistically significant, it was not included in the final model.

The stability of the results was tested in sensitivity analyses, in which one country from the HYENA study was removed in turn from analyses.

As women are prone to hormonal disturbances with menopause, sensitivity analyses were also carried out for women under and above 50 years of age separately (*N* = 286 and *N* = 459 respectively).

Some evidence suggests that annoyance may be on the causal pathway between noise exposure and hypertension [38, 39] or saliva cortisol [40]. Noise sensitivity can be conceptualized as a modifier or mediator of the effects of noise exposure on the outcome measured [41]. Thus, these factors have been both included in an additional model as covariates, and regarded as factor of interest instead of noise levels. However, when they were included as covariates, they did not modify the results, so these factors were not included in the final models.

Coefficients and 95% CIs were calculated to show the average variation in outcomes for a 10-dB(A) increase in noise. All statistical analyses were performed with SAS software V. 9.4 (SAS Institute, Cary NC) using the GLM procedure.

## Results

**Fig. 2:**
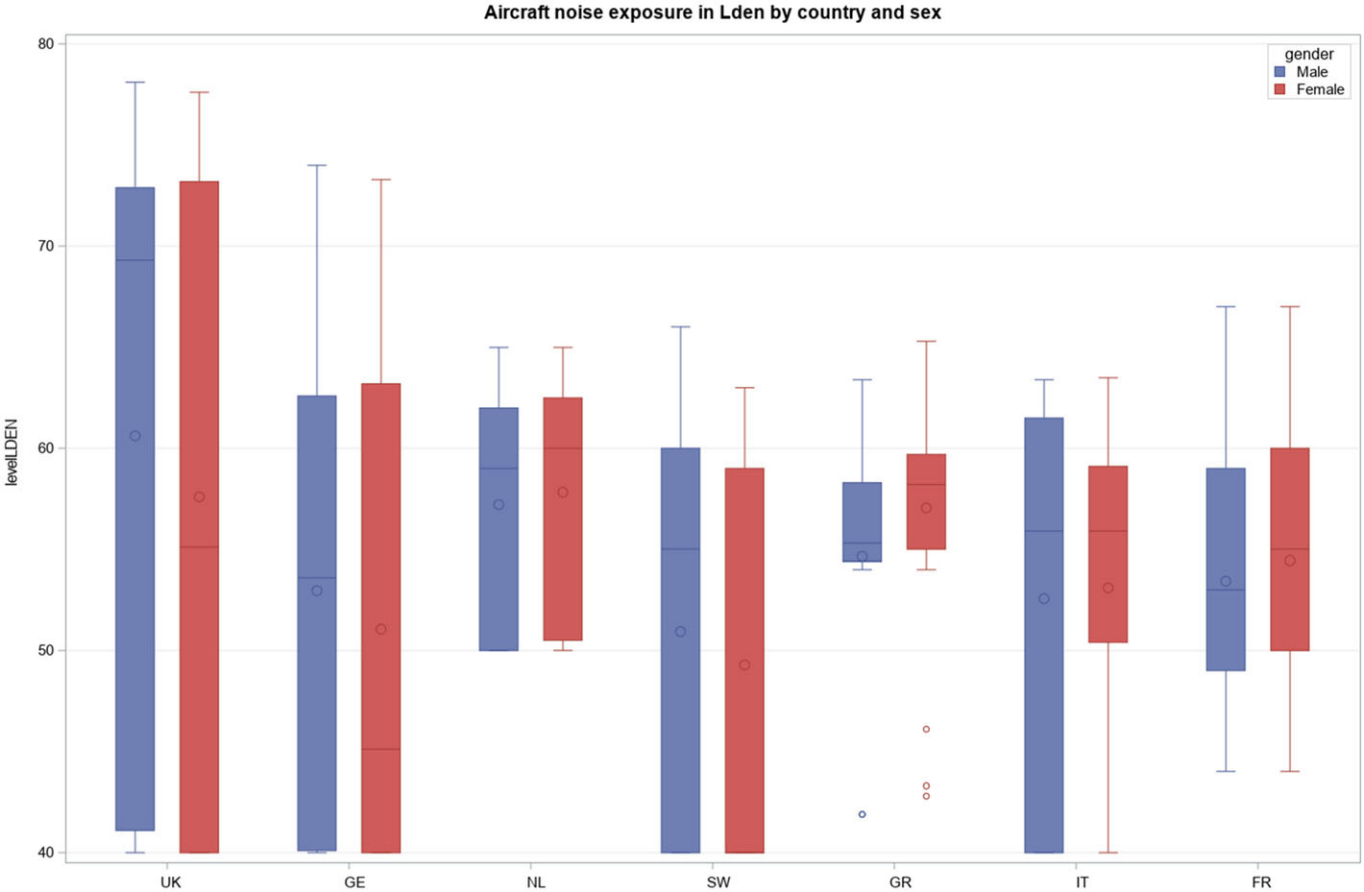
L_den_ levels by country and sex. (UK: United Kingdom; GE: Germany; NL: The Netherlands; SW: Sweden; GR: Greece; IT: Italy; FR: France)

Participation rates differed between countries, from approximately 30% in France, Germany, Italy, and the UK to 46% in the Netherlands, 56% in Greece, and 78% in Sweden. Figure 2 shows noise exposure levels by country and gender. The four noise indicators were highly correlated (correlation coefficients between 0.80 and 0.99). Participants from the UK were more likely to be exposed to higher noise levels compared to participants from other countries (*p* < 0.001). Characteristics of the study population, stratified by categories of noise exposure in terms of L_den_ are presented in the Table 1. Few differences appeared between noise categories. They are related to alcohol intake in men (*p* = 0.006) and physical activity in women (*p* = 0.003).

**Table 1:** HYDE study population characteristics stratified by aircraft noise categories by sex sub-groups (L_den_)

	MALE						FEMALE					
	L_den_						L_den_					
	< 50 dB(A)	50–54 dB(A)	55–59 dB(A)	≥60 dB(A)	N total	p(χ^2^) or p > F	< 50 dB(A)	50–54 dB(A)	55–59 dB(A)	≥60 dB(A)	N total	p(χ^2^) or p > F
Age (mean ± SD)	54.9 (13.0)	54.1 (14.4)	53.8 (14.6)	55.9 (12.3)	**555**	**0.606**	50.8 (13.7)	52.7 (14.6)	52.5 (15.7)	54.6 (14.5)	**745**	**0.083**
BMI (mean ± SD)	26.8 (4.0)	26.7 (5.1)	27.5 (5.0)	27.4 (4.4)	**555**	**0.369**	25.7 (5.2)	25.6 (5.4)	26.2 (5.8)	27.0 (5.6)	**745**	**0.066**
Alcohol (units/week)^1^						**0.006**						**0.370**
teetotaller	5.4	2.5	5.2	7.0	**112**		8.1	7.4	9.1	9.9	**257**	
1–7	15.0	14.2	9.4	14.6	**295**		13.6	9.1	11.0	13.8	**354**	
8–14	6.5	4.0	2.3	2.9	**87**		4.2	4.0	3.0	3.8	**111**	
> 14	2.5	2.2	2.3	4.0	**61**		1.1	0.3	1.1	0.7	**23**	
Smoker habits (units/day)^1^						**0.650**						**0.417**
non-smoker	11.5	11.2	7.9	12.8	**241**		13.3	12.0	14.0	16.5	**415**	
exsmoker	13.0	7.8	7.4	10.6	**215**		8.1	3.9	4.8	6.4	**173**	
0–10	2.9	1.4	1.8	1.8	**44**		3.1	3.0	3.1	2.8	**89**	
11–20	1.3	1.1	1.3	1.4	**28**		1.9	1.2	1.3	1.9	**47**	
> 20	0.7	1.4	0.9	1.8	**27**		0.5	0.8	0.9	0.5	**21**	
Education^1^						**0.515**						**0.098**
1st qrt	10.8	7.8	6.9	11.4	**204**		6.7	7.4	8.3	9.8	**240**	
2nd qrt	5.2	3.2	3.8	4.3	**92**		5.9	3.6	4.0	7.1	**154**	
3rd qrt	4.5	4.5	2.7	6.3	**100**		4.8	4.0	3.9	4.6	**129**	
4rd qrt	8.8	7.4	6.0	6.5	**159**		9.4	5.8	7.9	6.7	**222**	
Physical activity^1^						**0.596**						**0.003**
No or little	14.4	11.5	9.9	16.0	**288**		12.6	10.2	14.0	17.9	**407**	
Regular	15.0	11.4	9.4	12.4	**267**		14.2	10.6	10.2	10.3	**338**	
Total	**163**	**127**	**107**	**158**	**555**		**200**	**155**	**180**	**210**	**745**	

^1^ Percentage of participants

There was no statistically significant gender difference for the cortisol levels (Table 2): geometric means for morning levels were slightly higher for men compared to women (20.3 nmol. L^− 1^ and 19.9 nmol. L^− 1^ respectively), whereas geometric means for evening levels were slightly lower for men compared to women (3.9 nmol. L^− 1^ and 4.0 nmol. L^− 1^ respectively). Men showed a slightly higher geometric mean for the variation per hour than women (1.4 nmol. L^− 1^.H^− 1^ and 1.2 nmol. L^− 1^.H^− 1^ for men and women respectively.) The geometric means for the average relative-variation per hour in men and in women were statistically and significantly different (0.07 and 0.06 respectively (*p* = 0.037)).

**Table 2:** Geometric means (Standard Deviation) for cortisol outcomes by country and by sex-subgroups

	MALE					FEMALE				
	N	Morning level (nmol. L^−1^)	Evening level (nmol. L^−1^)	Variation per hour (nmol. L^−1^.H^−1^)	Relative-variation per hour	N	Morning level (nmol. L^−1^)	Evening level (nmol. L^− 1^)	Variation per hour (nmol. L^− 1^.H^− 1^)	Relative-variation per hour
UK	**35**	17.2 (2.0)	2.5 (2.1)	1.0 (2.2)	0.06 (1.5)	**31**	18.0 (1.8)	2.2 (2.6)	1.1 (2.1)	0.06 (1.3)
GE	**28**	20.5 (1.8)	2.6 (2.2)	1.0 (2.2)	0.05 (1.8)	**39**	15.7 (1.9)	1.9 (1.7)	0.9 (2.8)	0.06 (1.6)
NL	**29**	16.0 (1.9)	2.6 (2.3)	1.0 (2.1)	0.06 (1.6)	**28**	18.2 (1.6)	1.9 (1.9)	1.1 (1.9)	0.06 (1.4)
SW	**31**	18.7 (1.7)	4.4 (2.1)	0.8 (2.1)	0.04 (1.6)	**43**	17.3 (1.8)	2.9 (2.3)	0.9 (1.9)	0.05 (1.2)
GR	**14**	12.6 (2.5)	1.7 (2.3)	0.8 (2.7)	0.06 (1.6)	**31**	8.4 (2.4)	1.7 (2.3)	0.5 (2.8)	0.05 (1.6)
IT	**22**	20.5 (1.6)	2.9 (2.1)	1.2 (1.8)	0.06 (1.3)	**28**	17.3 (1.9)	3.4 (2.4)	0.7 (3.2)	0.04 (2.0)
FR	**396**	21.4 (1.9)	4.4 (2.2)	1.6 (2.9)	0.07 (2.0)	**545**	21.9 (1.9)	5.0 (2.2)	1.4 (3.1)	0.07 (2.1)
TOTAL	**555**	**20.3 (1.9)**	**3.9 (2.3)**	**1.4 (2.8)**	**0.07 (1.9)**	**745**	**19.9 (2.0)**	**4.0 (2.4)**	**1.2 (3.0)**	**0.06 (2.0)**

Crude and adjusted linear regression coefficients after exponentiation for each of the cortisol outcomes (morning and evening cortisol levels, variation per hour, and relative-variation per hour) in relation to aircraft noise levels are shown in Table 3. Analyses were performed for each noise indicator separately. No statistically significant association was found in men, for any of the cortisol outcomes, or noise indicators, both for univariate and multivariate analyses. Statistically significant crude estimates were shown in women only for the L_night_ in relation to the evening level, and for all the noise indicators in relation to the relative variation. For multivariate analyses, aircraft noise levels were not related to the morning concentration of cortisol, but were associated with an increase in evening cortisol concentration (exp(β) = 1.08, CI95% 1.00–1.16 for a 10-dB(A) increase in LA_eq,16h_; exp.(β) = 1.09, CI95% 1.01–1.18 for a 10-dB(A) increase in L_den_; exp.(β) = 1.11, CI95% 1.02–1.20 for a 10-dB(A) increase in L_night_ – while the relation between evening level and LA_eq, 24h_ was borderline significant exp.(β) = 1.08, CI95% 1.00–1.17 per 10-dB(A) increase) in women. For cortisol variation per hour, an association at the borderline of the statistical significance was found with L_night_ suggesting a 11%-decrease in the average difference between both samplings (exp(β) = 0.89, CI95% 0.80–1.00 per 10-dB(A) increase). Considering the relative-variation per hour, statistically significant associations were seen for a decrease of the relative difference between both samplings, whatever the noise indicator (a decrease of 8 to 11% according to the considered noise indicator). The strongest association was found for the L_night_ exposure (exp(β) = 0.89, CI95% 0.83–0.96 per 10-dB(A) increase).

**Table 3:** Linear regression coefficient after exponentiation for the relation between cortisol outcomes and aircraft noise levels

		MALE								FEMALE							
		Morning level (nmol. L^−1^)		Evening level (nmol. L^−1^)		Variation per hour (nmol. L^−1^.H^−1^)		Relative-variation per hour		Morning level (nmol. L^− 1^)		Evening level (nmol. L^− 1^)		Variation per hour (nmol. L^− 1^.H^− 1^)		Relative-variation per hour	
		exp(β)	CI95%	exp(β)	CI95%	exp(β)	CI95%	exp(β)	CI95%	exp(β)	CI95%	exp(β)	CI95%	exp(β)	CI95%	exp(β)	CI95%
Crude estimates	**L**_**Aeq,16h**_	0.96	(0.90–1.02)	0.99	(0.91–1.07)	0.90	(0.82–1.00)	0.95	(0.88–1.01)	1.00	(0.94–1.07)	1.04	(0.96–1.13)	0.92	(0.83–1.01)	**0.91**	**(0.86–0.97)**
	**L**_**Aeq,24h**_	0.95	(0.88–1.02)	0.98	(0.9–1.070)	0.90	(0.80–1.00)	0.95	(0.88–1.02)	1.02	(0.95–1.09)	1.06	(0.97–1.15)	0.93	(0.84–1.04)	**0.92**	**(0.86–0.98)**
	**L**_**den**_	0.96	(0.89–1.03)	1.00	(0.91–1.09)	0.90	(0.81–1.01)	0.94	(0.88–1.01)	1.01	(0.95–1.08)	1.08	(1.00–1.18)	0.91	(0.82–1.02)	**0.91**	**(0.85–0.97)**
	**L**_**night**_	1.01	(0.94–1.08)	1.08	(1.00–1.17)	0.99	(0.89–1.09)	0.98	(0.92–1.05)	1.04	(0.97–1.10)	**1.24**	**(1.15–1.34)**	0.97	(0.87–1.07)	**0.93**	**(0.88–0.99)**
Adjusted models^a^	**L**_**Aeq,16h**_	0.99	(0.92–1.06)	1.04	(0.95–1.12)	0.95	(0.86–1.06)	0.97	(0.90–1.04)	1.04	(0.98–1.10)	**1.08**	**(1.00–1.16)**	0.95	(0.86–1.06)	**0.92**	**(0.86–0.98)**
	**L**_**Aeq,24h**_	0.98	(0.91–1.05)	1.03	(0.94–1.12)	0.94	(0.84–1.06)	0.96	(0.89–1.04)	1.05	(0.98–1.12)	1.08	(1.00–1.17)	0.96	(0.86–1.08)	**0.92**	**(0.85–0.98)**
	**L**_**den**_	0.99	(0.92–1.06)	1.04	(0.95–1.14)	0.95	(0.85–1.06)	0.96	(0.89–1.04)	1.03	(0.97–1.10)	**1.09**	**(1.01–1.18)**	0.93	(0.84–1.04)	**0.90**	**(0.84–0.97)**
	**L**_**night**_	1.00	(0.93–1.08)	1.05	(0.97–1.15)	0.95	(0.85–1.06)	0.95	(0.88–1.02)	1.00	(0.94–1.07)	**1.11**	**(1.02–1.20)**	**0.89**	**(0.80–1.00)**	**0.89**	**(0.83–0.96)**

^a^ adjusted for country, alcohol intake, smoking habits, physical activity, education level, age and BMI (statistically significant values in bold)

No obvious differences were observed when noise annoyance or noise sensitivity were included in the model. When these factors were included in the models as factor of interest instead of noise levels, results did not show statistically significant association with cortisol outcomes, except an association between aircraft noise annoyance and the relative variation of cortisol in women only (exp(β) = 0.82, CI95% 0.72–0.93 for highly annoyed women compared to not annoyed women).

When analyses were carried out removing one country from the HYENA study in turn from the study population, results were similar to those found for the whole study population (See Additional file 1: Table S1).

Analyses carried for women under or above 50 years of age showed statistically significant associations between aircraft noise exposure and cortisol outcomes in women under 50, whereas no statistically significant association was found in women 50 and older (See Additional file 1: Table S2).

Finally, the results of the sensitivity analyses with the adjusted cortisol levels (results not shown) to overcome differences in the cortisol determination method (ELISA versus RIA method) were similar to the results in Table 3.

## Discussion

This study provides some support for the hypothesis that psychological stress induced by aircraft noise exposure, results in disturbed cortisol regulation, with an increase in evening cortisol levels and a flattening of the usual (absolute or relative) variation per hour. The results of the present study are partly in line with those found based on the DEBATS participants only [25], where similar conclusions were drawn, but in that study no difference were shown between gender subgroups. Considering the HYENA participants only, statistically significant associations were found only in women, but for an increase in the morning cortisol levels [17]. The HYDE pooled-analyses, providing a greater statistical power, found statistically significant associations between aircraft noise exposure and modification of the cortisol stress-hormone secretion for women only. Moreover, the main finding of this study was the statistically significant associations between noise levels and noise annoyance and the average relative-variation per hour.

The use of relative-variation per hour contributes to a new approach to analysis of cortisol in relation to noise exposure. It also allows for potential measurement differences between HYENA and DEBATS related to sampling and laboratory analysis: the ELISA (enzyme-linked immunosorbent) method in the DEBATS study and the RIA (radioimmunoassay) method in the HYENA. For morning and evening cortisol levels, the use of the linear relationship between ELISA and RIA methods [35] makes levels of cortisol concentrations directly comparable for the HYDE study. Regression conclusions were similar to those presented in Table 3, carried out without the use of this equation.

Although procedures in samplings were similar, differences occurred in the morning sampling which was requested to be collected 30 min after awakening in HYENA, and directly at awakening in DEBATS. It has been shown that cortisol levels rapidly rise by 50–75% within the first 30 min after awakening, and remain elevated for the next 30 min after the peak. This pattern in cortisol secretion has been shown to be independent of time of awakening, sleep quality, sleep duration, and age [42]. However, it seems quite difficult to define precisely when the awakening occurred. If morning cortisol samples were collected before the cortisol peak in the DEBATS study, it is likely that these cortisol levels were lower than the peak level. However, this measurement bias is independent from the noise levels, and could stand for a non-differential measurement error. Thus, it could have led to biased results toward the null value and could explain the fact that no association was observed for morning concentrations. Despite this, a statistically significant association was still observed for morning-evening cortisol variation and average relative cortisol variation per hour.

The present study found statistically significant associations between aircraft noise levels and cortisol outcomes only in women. Even if they were closer to 1, the estimates for males were not so different from that of females, especially for the relative variation per hour. This can either be explained by the fact that the men sample was smaller than the female study sample or by the fact that there was no association in men.

These results are in line with those showing that women were more sensitive to stressors in general [43, 44]. But they are not in line with those of previous studies considering gender-differences and showing statistically significant associations between aircraft or traffic noise exposure and hypertension only in men [5, 36, 45]. As statistically significant associations in women were stronger considering aircraft noise levels during the night with the L_night_, the hypothesis that women have a higher susceptibility to noise during sleep could be relevant. Recent research has pointed out different physiological characteristics in both men and women, leading to differences in the pathogenesis of cardiovascular diseases. Indeed, several studies on the effects of traffic noise exposure on the risk of hypertension showed stronger associations for men [1, 46–48]. This interaction could also explain the findings of the present study concerning the results of analyses in women under and over 50 years of age. Indeed, the associations were stronger in women under 50 years of age, while there was no statistically significant relationship in women over 50 years of age. After 50 years of age, with the disappearance of oestrogens, women’s hormonal systems tend to get closer to those of men, thus could explain that the results were almost similar in men and in women after 50 years of age in this study.

Statistically significant associations in women were stronger for aircraft noise levels during the night. These results support the hypothesis that susceptibility to noise would be higher during the night [49]. Sleep duration could be regarded as a confounder or as a mediator in the relation between aircraft noise exposure and the cortisol secretion [40, 50], since night-time noise can impact on sleep latency and induce early morning awakenings. Nevertheless, when this variable was included in the models, the results remained very similar, thus excluding the role of confounder or of mediator of sleep duration in the association between aircraft noise levels and cortisol secretion. As restrictions in night traffic concerned every airport except London’s Heathrow and Amsterdam Schiphol, we added an interaction term between country and noise exposure levels in multivariate models. Nevertheless, no substantial difference between countries was seen (results not shown). In sensitivity analyses, the results remained similar when the HYENA countries were removed one at a time from analyses. Associations were even stronger when the UK, the country with the highest noise exposure levels, was removed.

Selection bias cannot be excluded in the present study. The low response rate in most of the participating countries may be a potential weakness of this study. However, only minor differences were found between the characteristics of the participants and those of the nonresponders according to aircraft noise exposure categories [5, 36]. Aircraft noise levels were estimated at home address of each participant, but this may result in some exposure misclassification. It was not possible to take into account their noise exposure outside their home because no information was collected on time schedule and the work or leisure places of each participant. It is more likely that people are in their home at night, so night-time exposure is more likely to be close to the estimated noise level and may be the reason that the strongest associations were observed for L_night_. However, as the four noise indicators were highly correlated, this did not allow the effects of noise exposure during the day or at night to be disentangled.

When annoyance from aircraft noise or noise sensitivity were included as confounders in the models, no obvious differences were seen in results. These findings suggest that cortisol variations may be directly connected to aircraft noise exposure rather than mediated through noise annoyance and noise sensitivity.

Noise has been assumed to be a non-specific stress factor activating the autonomic nervous system and endocrine pathway [12, 51]. Stress is biologically distinguished by the secretion of hormones: catecholamines (norepinephrine, adrenaline) and cortisol [52]. The use of saliva cortisol in this study rather than blood cortisol or cortisol in the urine has been motivated by the fact that it is easy to measure in the general population, by its reliability, rapidity to sample, and non-invasiveness [53]. However, cortisol secretion can be influenced by season, time and week day of sampling [54], sex, age, BMI, physical activity, alcohol consumption, smoking, medication, high food intake, sleep quality, occupational activity, female hormonal contraception, and pregnancy [50] - all factors that affect hormonal balance [55]. All of these factors may moderate the association between aircraft noise exposure and cortisol secretion. Residual confounding bias cannot be excluded in this study but we have tried to minimize it by including all the information we have about these factors in the models.

The findings of the present study support a hypothesis that noise induces stress and are coherent with previous studies finding associations between aircraft noise exposure and hypertension or antihypertensive and anxiolytic medication intake [5, 36, 37]. There is evidence suggesting a role for cortisol in hypertension [56, 57], and other stated for an increase in the cortisol stress response responding to mental stress tasks, thus leading to incident hypertension [58].

## Conclusions

The present results support the hypothesis that exposure to aircraft noise, at night in particular, could make the HPA axis less flexible, especially in women, resulting in higher average values for the evening cortisol concentration, and therefore a flattening of the difference between morning and evening levels. The cause/effect relations and the biological process between noise exposure and HPA dysregulation are of major importance and need to be further elucidated.

## Supplementary information

**Additional file 1: Table S1.** Linear regression coefficient after exponentiation for the relation between cortisol outcomes and aircraft noise levels removing in turn one country from the HYENA study. **Table S2.** Linear regression coefficient after exponentiation for the relation between cortisol outcomes and aircraft noise levels in women under and above 50 years old.

## Data Availability

Not applicable.
