# Supplementary material for: Saliva cortisol in relation to aircraft noise exposure: pooled-analysis results from seven European countries
Source: Environ Health. 2019 Nov 27;18:102. doi: 10.1186/s12940-019-0540-0 (PMC6882169; doi:10.1186/s12940-019-0540-0)
Supplement: Supplementary file 1 — Additional file 1: Table S1. Linear regression coefficient after exponentiation for the relation between cortisol outcomes and aircraft noise levels removing in turn one country from the HYENA study. Table S2. Linear regression coefficient after exponentiation for the relation between cortisol outcomes and aircraft noise levels in women under and above 50 years old. [file 12940_2019_540_MOESM1_ESM.docx]

**Tables S1.** Linear regression coefficient after exponentiation for the relation between cortisol outcomes and aircraft noise levels removing in turn one country from the HYENA study.

|  | | **MALE** | | | | | | | | **FEMALE** | | | | | | | |
| --- | --- | --- | --- | --- | --- | --- | --- | --- | --- | --- | --- | --- | --- | --- | --- | --- | --- |
|  |  | **Morning level (nmol. L^-1^)** | | **Evening level(nmol. L^-1^)** | | **Variation per hour (nmol. L^-1^.H^-1^)** | | **Relative-variation per hour** | | **Morning level (nmol. L^-1^)** | | **Evening level(nmol. L^-1^)** | | **Variation per hour (nmol. L^-1^.H^-1^)** | | **Relative-variation per hour** | |
|  |  | exp(β) | CI95% | exp(β) | CI95% | exp(β) | CI95% | exp(β) | CI95% | exp(β) | CI95% | exp(β) | CI95% | exp(β) | CI95% | exp(β) | CI95% |
| Removing UK -66 people | **L_Aeq,16h_** | 0.98 | (0.91-1.06) | 1.02 | (0.93-1.12) | 0.93 | (0.83-1.05) | 0.95 | (0.88-1.03) | 1.02 | (0.95-1.09) | 1.06 | (0.97-1.15) | 0.91 | (0.81-1.02) | **0.90** | **(0.83-0.96)** |
|  | **L_Aeq,24h_** | 0.97 | (0.89-1.05) | 1.01 | (0.92-1.12) | 0.91 | (0.80-1.04) | 0.95 | (0.87-1.03) | 1.03 | (0.95-1.11) | 1.06 | (0.97-1.16) | 0.92 | (0.81-1.04) | **0.89** | **(0.82-0.97)** |
|  | **L_den_** | 0.98 | (0.90-1.06) | 1.03 | (0.94-1.14) | 0.92 | (0.81-1.05) | 0.95 | (0.87-1.03) | 1.01 | (0.94-1.08) | 1.07 | (0.98-1.17) | 0.88 | (0.78-1.00) | **0.88** | **(0.81-0.95)** |
|  | **L_night_** | 0.98 | (0.90-1.07) | 1.05 | (0.95-1.16) | 0.91 | (0.80-1.04) | 0.93 | (0.85-1.02) | 0.97 | (0.90-1.05) | **1.09** | **(1.00-1.19)** | **0.84** | **(0.74-0.94)** | **0.86** | **(0.79-0.93)** |
| Removing GE -67 people | **L_Aeq,16h_** | 0.98 | (0.91-1.06) | 1.07 | (0.98-1.17) | 0.94 | (0.84-1.05) | 0.96 | (0.89-1.03) | 1.05 | (0.98-1.12) | **1.09** | **(1.00-1.18)** | 0.95 | (0.85-1.06) | **0.91** | **(0.84-0.97)** |
|  | **L_Aeq,24h_** | 0.97 | (0.90-1.05) | 1.07 | (0.97-1.17) | 0.93 | (0.82-1.05) | 0.95 | (0.88-1.03) | 1.06 | (0.99-1.14) | **1.09** | **(1.00-1.19)** | 0.96 | (0.85-1.08) | **0.90** | **(0.83-0.98)** |
|  | **L_den_** | 0.98 | (0.91-1.06) | 1.08 | (0.99-1.19) | 0.93 | (0.83-1.05) | 0.95 | (0.88-1.03) | 1.04 | (0.97-1.12) | **1.10** | **(1.01-1.20)** | 0.92 | (0.82-1.04) | **0.89** | **(0.82-0.96)** |
|  | **L_night_** | 1.00 | (0.92-1.08) | **1.10** | **(1.00-1.20)** | 0.93 | (0.83-1.05) | 0.94 | (0.86-1.01) | 1.00 | (0.94-1.08) | **1.12** | **(1.02-1.22)** | **0.88** | **(0.78-0.99)** | **0.88** | **(0.81-0.95)** |
| Removing NL-57 people | **L_Aeq,16h_** | 0.99 | (0.92-1.06) | 1.03 | (0.95-1.12) | 0.96 | (0.86-1.07) | 0.97 | (0.91-1.04) | 1.04 | (0.98-1.11) | **1.09** | **(1.01-1.17)** | 0.95 | (0.86-1.06) | **0.92** | **(0.85-0.98)** |
|  | **L_Aeq,24h_** | 0.98 | (0.91-1.05) | 1.02 | (0.94-1.12) | 0.95 | (0.84-1.06) | 0.97 | (0.90-1.05) | 1.05 | (0.98-1.12) | **1.09** | **(1.01-1.19)** | 0.96 | (0.86-1.08) | **0.91** | **(0.85-0.98)** |
|  | **L_den_** | 0.99 | (0.91-1.06) | 1.04 | (0.95-1.13) | 0.95 | (0.85-1.07) | 0.97 | (0.90-1.04) | 1.03 | (0.97-1.10) | **1.10** | **(1.01-1.19)** | 0.93 | (0.83-1.04) | **0.90** | **(0.84-0.97)** |
|  | **L_night_** | 1.00 | (0.93-1.07) | 1.06 | (0.97-1.15) | 0.95 | (0.85-1.06) | 0.95 | (0.88-1.03) | 1.01 | (0.94-1.08) | **1.11** | **(1.02-1.20)** | 0.90 | (0.80-1.00) | **0.89** | **(0.83-0.96)** |
| Removing SW-74 people | **L_Aeq,16h_** | 0.99 | (0.92-1.07) | 1.04 | (0.96-1.14) | 0.95 | (0.85-1.07) | 0.96 | (0.89-1.04) | 1.04 | (0.97-1.11) | 1.08 | (0.99-1.17) | 0.94 | (0.84-1.05) | **0.91** | **(0.84-0.97)** |
|  | **L_Aeq,24h_** | 0.98 | (0.90-1.06) | 1.03 | (0.94-1.14) | 0.94 | (0.83-1.06) | 0.96 | (0.89-1.04) | 1.05 | (0.98-1.13) | 1.08 | (0.99-1.18) | 0.95 | (0.84-1.08) | **0.90** | **(0.83-0.98)** |
|  | **L_den_** | 0.99 | (0.91-1.07) | 1.05 | (0.96-1.15) | 0.95 | (0.84-1.07) | 0.96 | (0.89-1.04) | 1.03 | (0.96-1.11) | **1.09** | **(1.00-1.19)** | 0.92 | (0.82-1.04) | **0.89** | **(0.83-0.97)** |
|  | **L_night_** | 1.00 | (0.93-1.08) | 1.07 | (0.98-1.17) | 0.95 | (0.84-1.07) | 0.95 | (0.88-1.03) | 1.01 | (0.94-1.08) | **1.11** | **(1.02-1.20)** | 0.89 | (0.80-1.00) | **0.89** | **(0.82-0.95)** |
| Removing GR-45 people | **L_Aeq,16h_** | 0.99 | (0.93-1.06) | 1.03 | (0.95-1.12) | 0.96 | (0.86-1.06) | 0.96 | (0.90-1.03) | 1.03 | (0.97-1.10) | **1.09** | **(1.01-1.17)** | 0.95 | (0.86-1.06) | **0.92** | **(0.86-0.98)** |
|  | **L_Aeq,24h_** | 0.98 | (0.91-1.06) | 1.02 | (0.93-1.11) | 0.94 | (0.84-1.06) | 0.96 | (0.89-1.04) | 1.04 | (0.98-1.12) | **1.09** | **(1.01-1.19)** | 0.96 | (0.86-1.08) | **0.92** | **(0.86-0.99)** |
|  | **L_den_** | 0.99 | (0.92-1.07) | 1.04 | (0.95-1.13) | 0.95 | (0.85-1.06) | 0.96 | (0.89-1.03) | 1.03 | (0.96-1.10) | **1.10** | **(1.02-1.19)** | 0.93 | (0.83-1.04) | **0.91** | **(0.84-0.97)** |
|  | **L_night_** | 1.00 | (0.93-1.08) | 1.05 | (0.96-1.15) | 0.95 | (0.85-1.06) | 0.95 | (0.88-1.03) | 1.01 | (0.94-1.07) | **1.11** | **(1.03-1.20)** | 0.90 | (0.81-1.00) | **0.90** | **(0.83-0.96)** |
| Removing IT-50 people | **L_Aeq,16h_** | 1.00 | (0.93-1.08) | 1.05 | (0.97-1.14) | 0.96 | (0.86-1.08) | 0.96 | (0.89-1.03) | 1.05 | (0.99-1.12) | **1.10** | **(1.02-1.19)** | 0.96 | (0.86-1.07) | **0.91** | **(0.85-0.98)** |
|  | **L_Aeq,24h_** | 1.00 | (0.92-1.08) | 1.05 | (0.96-1.14) | 0.95 | (0.85-1.07) | 0.96 | (0.89-1.04) | **1.07** | **(1.00-1.14)** | **1.11** | **(1.02-1.20)** | 0.97 | (0.87-1.09) | **0.91** | **(0.85-0.98)** |
|  | **L_den_** | 1.00 | (0.93-1.08) | 1.06 | (0.97-1.16) | 0.96 | (0.85-1.08) | 0.96 | (0.89-1.03) | 1.05 | (0.98-1.12) | **1.11** | **(1.02-1.20)** | 0.94 | (0.84-1.05) | **0.90** | **(0.84-0.97)** |
|  | **L_night_** | 1.01 | (0.94-1.09) | 1.07 | (0.98-1.17) | 0.96 | (0.85-1.08) | 0.95 | (0.88-1.03) | 1.02 | (0.95-1.08) | **1.12** | **(1.03-1.22)** | 0.90 | (0.81-1.01) | **0.89** | **(0.83-0.96)** |

adjusted for alcohol intake, smoking habits, physical activity, education level, age and BMI (Statistically significant values in bold)

Table S2: Linear regression coefficient after exponentiation for the relation between cortisol outcomes and aircraft noise levels in women under and above 50 years old

|  | **FEMALE <50 years old (N = 286)** | | | | | | | | **FEMALE ≥50 years old (N = 459)** | | | | | | | |
| --- | --- | --- | --- | --- | --- | --- | --- | --- | --- | --- | --- | --- | --- | --- | --- | --- |
|  | **Morning level (nmol. L^-1^)** | | **Evening level(nmol. L^-1^)** | | **Variation per hour (nmol. L^-1^.H^-1^)** | | **Relative-variation per hour** | | **Morning level (nmol. L^-1^)** | | **Evening level (nmol. L^-1^)** | | **Variation per hour (nmol. L^-1^.H^-1^)** | | **Relative-variation per hour** | |
|  | exp(β) | CI95% | exp(β) | CI95% | exp(β) | CI95% | exp(β) | CI95% | exp(β) | CI95% | exp(β) | CI95% | exp(β) | CI95% | exp(β) | CI95% |
| **LA_eq,16h_** | 1.11 | (1.00;1.25) | **1.21** | **(1.05;1.40)** | 0.93 | (0.76;1.14) | **0.84** | **(0.73;0.96)** | 1.01 | (0.93;1.09) | 1.02 | (0.93;1.11) | 0.95 | (0.83;1.07) | 0.94 | (0.87;1.02) |
| **LA_eq,24h_** | **1.18** | **(1.03;1.34)** | **1.28** | **(1.08;1.51)** | 0.95 | (0.76;1.20) | **0.81** | **(0.70;0.95)** | 1.01 | (0.93;1.10) | 1.01 | (0.92;1.11) | 0.95 | (0.83;1.09) | 0.94 | (0.87;1.02) |
| **L_den_** | 1.12 | (0.99;1.26) | **1.26** | **(1.08;1.47)** | 0.91 | (0.73;1.12) | **0.81** | **(0.70;0.93)** | 1.00 | (0.92;1.09) | 1.01 | (0.92;1.12) | 0.93 | (0.81;1.06) | 0.93 | (0.86;1.01) |
| **L_night_** | 1.06 | (0.95;1.18) | **1.24** | **(1.07;1.43)** | 0.84 | (0.69;1.03) | **0.80** | **(0.70;0.91)** | 0.97 | (0.89;1.06) | 1.03 | (0.93;1.13) | 0.89 | (0.78;1.03) | 0.92 | (0.84;1.00) |
